# The Significance and Diagnosis of Adenoids

**Published:** 1911-10-15

**Authors:** Wm. D. Sumpter

**Affiliations:** Nashville, Tenn.


### THE SIGNIFICANCE AND DIAGNOSIS OF ADENOIDS.

BY WM. D. SUMPTER, M.D., NASHVILLE, TENN.

Read before the Tennessee State Dental Association, May 24th, 1911.

Hypertrophy of normal lymphoid tissue in the post-nasal space constitutes a condition known as adenoids. This hyperplasia may be congenital; heredity seems to play a part in its etiology and often it is a family characteristic—several children of the same household being affected. The exciting causes are frequent nasal catarrh, sore throat, the eruptive diseases, diphtheria, whooping cough etc. The location of adenoids may be confined to the vault of the pharynx, or diffused over the posterior and lateral walls, even packing Rosenmuller's fossa, and adhering to the Eustachian tubes; their size may be small or sufficiently large to fill the post-nasal space; their shape presents an aggregated mass, or thickly scattered elevations, and when confined to the vault there is usually a median cleft with two bands extending to the posterior pillars of the fauces; their structure varies from vascular, spongy and soft bodies in infancy to more fibrous organization in older children;—their frequency is greatest in children from 6 to 15 years of age, although their removal has been necessary from early months of infancy to thirty years of age. In healthy children 1 to 9 per cent. are affected, and in deaf mutes 50 to 73 per cent.

The significance of adenoids is not fully appreciated by the dental or medical professions as evidenced by the inspection of school children, whose parents have not been advised by their dentists or family physicians of the existing conditions, and the grave results which may occur—Holt says, "This is a very common condition and one much neglected by the general practitioner. It is the source of more discomfort and the origin of more minor ailments than almost any other pathological condition of childhood." We should weigh well such a statement from the most eminent specialist on diseases of children in America. Many text-books on surgery make no mention of the subject and careful search in the text-books of two eminent dental surgeons reveals no discussion whatever of adenoids in works entitled Surgery of the Mouth and Jaw. I, therefore, wish to submit for the consideration of your association the importance of fully realizing the significance of adenoids, that you may be watchful and prevent the ill results which follow undiagnosed obstruction in the posterior nares. The serious consequences of obstructed nasal breathing are indeed worthy of our conscientious consideration whenever we look into the face or mouth of children under our care. Adenoid facial conformation and expression lasts through life, unless early removal is resorted to. Deformity of the superior maxillary bone occurs, and if the growth is present when the permanent teeth appear the canine teeth are pushed forward out of alignment, the incisors begin to protrude, are twisted on themselves, their posterior surfaces facing each other; the teeth are often irregular, and not in proper opposition. The nose broadens at its root, while the nostrils narrow and are pinched; the turbinates are poorly developed; the accessory sinuses of the nose are in an undeveloped condition, and the septum of the nose shows deviation. The mouth is held half open, the upper lip becomes short, and thick, the hard palate assumes an arched dome shape or decided V-shape. The general appearance of the countenance may be hatchet-faced. Other symptoms of mouth breathing appear as stupid expression of the face with drooping eyelids, snoring, defective speech, and almost constant catarrhal secretions which being swallowed induce indigestion. Anaemia, headache, cough, neuresis, mental dullness, nervousness by day and night terrors, even chorea and epilepsy have been caused by the obstruction. Spasmodic croup is said to be associated with adenoids in the majority of cases. Many of these conditions have been absolutely alleviated by removal of the growth; children at the foot of their classes have forged to the head after operation. Ballenger says, "If the removal of the growth is delayed until the individual has practically attained full growth, the mind will rarely develop as it would had it been removed."

The epipharyngeal mucous membrane is almost always in an acute or chronic state of inflammation from the presence of adenoids and produces serious effect on the ears by its contiguous relation to the Eustachian tube and middle ear, and adenoids are conceded to be the most frequent cause of otitis media. McBride and Turner analized 307 cases; 255 had ear complications of which 144 were suppurative and 111 were more or less deaf. Mastoid disease arising from middle ear disease has often its origin from adenoids.

The cervical lymphatic glands may become enlarged and meningitis simple or tubercular may result. In 945 cases collected by Lewin 5 per cent. were tuberculous. So important is this feature, added to the foregoing pathology that many of the larger cities require inspection and operation on all school children with affected tonsils or adenoids before they can continue in attendance. It is to be hoped the day may soon come, when with such inspection, including thorough examination of the teeth and eyes children in all schools may be blessed with the benefits of such prophylaxis. The suppurating condition of adenoids known as Thornwaldt's Disease, is in its influence on the health of the child of great concern and the effect on the constitution of the child is of such gravity that some one has said, "If you will show me a bow-legged man, I will show you one who has had adenoids in his infancy."

With a full consideration of the pathology that adenoid vegetations may induce we should feel that early diagnosis and prompt attention to the condition is imperative. The symptoms which have been given should suggest the existence of the pathological condition, although of course, as in any other disease they may not be prominent and the diagnosis be rendered obscure. Enlarged tonsils should be considered in the case. Ballenger says, "It may be stated as an almost universal rule that when the tonsils are hypertrophied, adenoids are also present. Conversely when adenoids are present the tonsils are not as often hypertrophied as only 30 per cent. of cases of adenoids showed enlargement of tonsils." How often does the dentist or physician associate or suspect adenoids in tonsillar enlargement?

Anterior rhinoscopy after shrinking the turbinates with cocain and suprarenal solution, will often reveal a pink or red mass at the back of the nares, and by using the laryngoscopic mirror in the posterior nares the growth may be seen, or the fringe of adenoids on the posterior wall of the pharynx, below the line of the soft palate, may be recognized. The easiest and absolutely certain method of diagnosis, however, is to explore the vault and sides of the pharynx by frapation. If adenoids are present, the finger determines accurately their existence and their location, so that in removing them, knowing their exact situation, a thorough operation can be done. A mouth gag is used to prevent biting and the index finger is easily passed under and behind the soft palate exploring the whole upper pharynx in a moment. The advice given by a specialist to push in the cheek with the thumb to guard against the teeth is not to be considered with the service rendered by the gag. The finger will almost invariably be covered with blood on removal, if adenoids are present. When such examination is made it is well to be prepared to operate on the case and if not vaseline should be used that the friction may not irritate the growth. For some time I have, before operating, entirely depended on this valuable method of diagnosis, which entails no uncertainty and feel that anyone can by its use easily clear up all doubtful cases. That fibrous tumors, malignant tuberculous and syphilitic growths etc., may occur in the pharynx must be borne in mind, but the firmness of fibroma, the history of malignant and other deposits should make the case clear in most instances of their rare occurrence in children. It should be remembered that adenoids associated with cleft palate are not uncommon and their removal should be effected before operation on the cleft.

After diagnosis of adenoids the growth should be promptly removed. That it subsides at puberty is not proved. The naso-pharynx expands at that time and may give relief in breathing but leaves the source of irritation not atrophied, but by continuous inflammation a menace to the ears, nose and throat. The thorough removal gives good results, however the process may have advanced, and as to deafness, nervousness, a guarded prognosis should always be given.

The technique of the operation is simple. I prefer the use of Gottstein's curette modified—and agree with many others that forceps is seldom indicated. After removal of the growth I examine the fossa with my finger wrapped in a layer of gauze which determines the thoroughness of the operation and removes any hanging shreds. This I consider necessary to insure results. The operation may be done with or without an anesthetic as the case demands. As an anesthetic ethyl chloride is ideal. In the scope of this paper I have not intended to discuss the operative technique or post-operative treatment. The most important consideration in my estimation is the early diagnosis and full understanding of the grave significance of adenoids as relates to the development of the children of our land—and contribute this plea to your profession's earnest attention and beg your assistance in the betterment and development of the generation that in a score of years will be our men and women whom we hope with health-giving advances in your profession and mine will stand unequalled as God's noblest work.

DISCUSSION.

Dr. J. D. Eby: Mr. President, I havn't anything particular to say other than the most hearty commendation of Doctor Sumpter's most timely and valuable paper. The per cent of irregularities of the teeth caused by adenoids, I could not state, but the ratio runs very high. Some of these irregularities are, open mouth breathing caused by the narrowness of the side and projection of the anterior teeth and the deflection of the nasal septum should be added to those the Doctor has described. It is a duty that the dentist owes to his patients that bring their children to him to have them examined for dental troubles, at least to recommend that they also have them examined for adenoids. If the adenoids are present we should recommend their removal.

Dr. A. D. Black: I believe the members of the medical profession, and the dental too, are beginning to realize more than they used to, but not yet as much as they ought to, the fact that the small irritations of children many times lead to very serious consequences. We have been making some very interesting tabulations and investigations of the condition of the children in our public schools in Chicago, and these tabulations are leading very decidedly to the conclusion that the child who has adenoids, the child who has teeth which are sufficiently uncomfortable to annoy it, the child who has any of what are generally considered minor defects, is a child who will not make proper progress in school, and will not make satisfactory development. In one of the largest school districts in New York City, a tabulation that has recently been made of five thousand school children, in which the report cards of those children were compared and all the children who were below grade were suspected after investigation it was found that among those children who were below grade that more than ninety per cent. had some defect of this kind. The largest percentage were defective in the teeth, some of them being abscessed and others in such a condition that the pulps were so nearly exposed so as to cause trouble. I remember that second in that list came adenoids in this percentage of children that had failed. These statistics are worth something to us and we ought to remember them in connection with this paper. We ought to see that the children that come into our offices shall go to a physician who is capable of making a thorough examination.

Dr. H. B. Johnson: I want to thank the Doctor for the excellent paper he has given us, and especially these illustrations. I believe from one of those illustrations that I have a case at home that has adenoids, as it is so much like one of the Doctor's pictures, that you would think he is a twin brother, and when I go back home I shall advise this father to have it looked into. I think we should be more of diagnosticians than most of us are, we should look further than the teeth, and if it should become absolutely necessary the dentist should be prepared to do an operation of this kind.

Dr. J. W. Bryan: All of us have seen this narrow, pinched expression, contracted and anxious look, irregular and protruding teeth, distended noses, a thoroughly anxious expression, you can tell them almost as you walk the streets. Just a few days ago a little girl twelve years old was in my office. I asked her if she did not suffer from trouble in breathing, and she said she did, and I told the party that was with her to tell her father that he ought to have her treated for adenoids. I expect if dentists should open up this line as specialists, there would be cause for a great deal of criticism. I am glad to say I know a specialist who can treat a case when I find one. I am glad these pictures have been shown to us, you will meet them on the street every day. In most cases after you see one of them you can diagnose the case by that narrow, contracted, pinched and uneasy expression just as it has been pictured on the board.

Dr. Johnson: I would like to ask the Doctor if he uses cocain as a local anesthetic in removing adenoids and how he applies it.

Dr. King: This paper calls to my mind a little patient that came into my hands recently. I tried to get the little fellow to come up this afternoon, but I could not prevail on him to come before the Association. A young man fell—came to me some time ago through his mother who was very much disturbed about his teeth, as he had a protrusion of all the upper anterior teeth and the arch was very much contracted. He was a typical mouth-breather, and he suffered as Dr. Sumpter described exactly. I suggested that she take the young man to a specialist and have the adenoids removed, as I was almost positive that they were present. The little fellow couldn't sleep very well at night, was very much emaciated and in very bad health. The adenoids were removed and since then I have been correcting the irregularity and protrusion of those teeth, spreading the upper and lower arches so that the teeth would have plenty of room. Since the operation the little fellow has gained ten pounds in weight, he does not look like the same child at all, his expression and carriage are different and in his grades at school are higher and he has increased in both mental and physical stature. I believe if we would pay more attention to these cases or irregularities as they come to us, we will find in a great many cases that adenoids complicate our work.

Dr. C. O. Rhea: I wish to thank the Doctor for his excellent paper he has given us, and I feel sure that we have all been benefited by it. The doctor told the chairman of the program committee that he was too busy to write a paper, and I told him that it was just from such people that we needed papers; the men who are not busy are not competent to write papers. If it is in order to do so I wish to move that Dr. Sumpter be made an honorary member of our society. Motion seconded and carried unanimously.

Dr. M. L. Rudolfh, of Clarksville: Dr. Sumpter let down the bars to the dental profession, but I would like to say to these dentists who would like to take up this branch of surgery that they should post themselves very carefully on the matter as I have seen one very serious hemorrhage from an operation, and I had rather the responsibilty should be on the surgeon than on myself.

Dr. M. D. Meadows: Many of us have claimed that dentistry is a distinct department of medicine and should be so treated, and so Dr. Sumpter is trying to turn us into lines that we have been too timid to take up, and we should be very thankful to him for his contribution to our program of to-day. The most interesting part of the discussion to me is the fact that Dr. Sumpter has in his clinic here exhibited adenoids from my child's mouth.

Another thing that I wanted to ask Dr. Sumpter to bring out was the probability of a recurrence of this growth. As a matter of fact the operation has been performed in my child's mouth twice, having previously been done by a specialist. I feel a sort of sympathy for the children of the State of Tennessee since this paper has been read here to-day, because dentists are not timid men as a class. Perhaps our experience will be in its effects like that of a few years ago when there was an advocacy of the use of castor oil and bitter aloes to drain out the alimentary canal, not for a few days, but for thirty days, and I think the price of castor oil advanced throughout the State of Tennessee, but I know I got good results from its use and I managed to get some patients to take castor oil daily for thirty days.

The operation does look simple to one who witnesses it, and I see no reason why dentistry should not take up the duty of removing adenoids if it choose to do so. Some dental surgeons make operations for cleft palate very successfully, and we may in time take up the operations. for cleft palates and adenoids as our rightful field for work. The most unfortunate complication, as suggested by Dr. Sumpter, is the susceptibility to the various diseases, such as diphtheria and other diseases. We should emphasize the fact that these examinations should be made early that the adenoids may be detected if they exist, not only from the standpoint of general health, but because of the results which we all know will follow as to irregularities of the teeth and because of the operations which are necessary for the correction of these irregularities. I am certainly glad that Dr. Sumpter has been with us and to know that he is an honorary member of our society, because we shall get some good work out of him in the future.

Dr. Leonard: I do not believe that the essayist was in earnest in advocating that the dentist should do these operations for adenoids. Of course it is not very far from the field that the dentist is in the habit of operating upon, but there are liable to be other complications that we would not be able to recognize, we should, however, be able to make a proper diagnosis to be able to refer patients to the proper specialist for treatment. I am one of those who believe that this is in the field of the rhinologist, and I think that all of these cases should be referred to the rhinologist. In some cases it is a simple operation, but the suggestion of the gentleman who spoke a while ago, Dr. Rudolph, of a recent hemorrhage that was the result of the removal of adenoids should make us careful, because the average dentist is hardly prepared to cope with the complications that might arise from the operation.

Dr. Sumpter: I am keenly appreciative of your courtesy of electing me to honorary membership in your society and I assure you that it has been a pleasure to be with you and take some part with you. Since 1895 I have been attempting in an humble way to help your students to be good dentists.

Referring to what Dr. Leonard said about my not being in earnest in recommending dentists to perform this operation, I would say that if I was a dentist I would take them out. I don't say you should. I didn't tell you to do it, I said you could do it, and you can. Some people would try to scare you out of this because they want to see the work turned over to the rhinologist. If a patient is brought to me with ear trouble, I will test the ear, and if there is anything the matter with it, I will turn him over to the rhinologist, as I don't treat ears. I know how to take out adenoids, and I expect now and then to get twenty-five or fifty dollars for doing it.

If you will notice the title of my paper, you will notice also that I have avoided discussing the operation and the diagnosis, I left that to you to take your choice of doing the work only for which you are held responsible, and of explaining to the family afterwards the other conditions which require medical treatment. When you perform this operation the patient spits much blood, in fact it looks like you had killed a hog right there on the spot, and you are likely to get blood all over everything. I have managed to control the blood by having a towel handy and as soon as my curette comes out it keeps the blood from scattering around. If you let the child drink ice water or ice tea, and hold it in the throat it will stop the bleeding.

There have been fatal results from this operation, but they will occur from anything. We have fatal results from the use of chloroform. But this question is not before us, the question of etiology is not before us, it is a question of diagnosing and understanding what adenoids mean. The general appearance of the child does almost indicate that he has adenoids. You can be almost as certain as you can that there are flies on the sugar that you left open yesterday. Then if you want to operate, do it. A good spray is dioxygen one part to four parts of water. I tried to get a clinic for you to-day, and I am still trying, if I can find a case for to-morrow I will do the operation for you.

I do a great proportion of these operations without an anesthetic. I take the curette and scratch out the adenoids and I do not use anesthetics unless the parents insist. Perhaps some will say because I do not use anesthetics that I am behind the times; I am behind that curette. If I use an anesthetic, I use ether or ethyl chloride; chloroform is dangerous. Cocain is not practicable for adenoids if the throat is inflamed.

